# SOCS3 as a potential driver of lung metastasis in colon cancer patients

**DOI:** 10.3389/fimmu.2023.1088542

**Published:** 2023-03-21

**Authors:** Xuejie Li, Zuyi Yang, Bi Chen, Lei Gu, Guoyan Tian, Xinbing Sui

**Affiliations:** ^1^Department of Pathology, The First Affiliated Hospital of Medical School of Zhejiang University, Hangzhou, China; ^2^Department of Hematology and Oncology, the Affiliated Hospital of Hangzhou Normal University, College of Medicine, Hangzhou Normal University, Hangzhou, China; ^3^School of Pharmacy, Hangzhou Normal University, Hangzhou, China; ^4^Key Laboratory of Elemene Class Anti-cancer Chinese Medicine of Zhejiang Province, Hangzhou Normal University, Hangzhou, China

**Keywords:** colon primary tumor, lung metastasis, SOCS3, macrophage, immune infiltration

## Abstract

**Background:**

The suppressor of cytokine signaling 3 (SOCS3) is the negative feedback regulator of the JAK-STAT signaling pathway. The purpose of our study was to investigate the SOCS3 status in colon primary tumor and lung metastasis and its relationship with macrophages.

**Methods:**

The SOCS3 expression pattern and its relationship with the immune response in pan-cancer was investigated using multiple methods. Samples and corresponding clinical information of 32 colon cancer patients with lung metastasis were collected, and the CD68, CD163, and SOCS3 status were conducted using immunohistochemistry (IHC). The relationship between SOCS3 status and macrophage markers was analyzed. Besides, we explored the molecular mechanisms of SOCS3 in lung metastasis *via* the TCGA database.

**Results:**

High SOCS3 expression was more inclined to poor prognosis and was positively correlated with main immune cell infiltration in almost each cancer type, especially in colon cancer. Compared with the colon primary tumor, lung metastasis harbored higher CD163 and SOCS3 expression, and high SOCS3 expression was more likely to be associated with high CD163 expression in lung metastasis. Besides, the exceptional differentially expressed genes in lung metastasis significantly enriched in immune responses and regulations.

**Conclusions:**

SOCS3 possessed value as a prognostic marker and target for immunotherapeutic intervention in different tumors and might be a potential target of tumor progression and tumor immunotherapy in colon cancer.

## Introduction

The JAK-STAT pathway is the center of signal transduction pathways to activate multiple cytokines, and its aberrant alterations involve the occurrence and development process of multiple malignant tumors and autoimmune diseases (1). Suppressors of cytokine signaling (SOCS) proteins are the negative physiologic regulator and compete with STATs by binding to the cytokine receptor to interfere with the signal transduction process (2). The SOCS family contains 8 intracellular protein members, including CIS and SOCS1-7 (3). These proteins all are centered on an SH2 domain, surrounded by a changeable length and sequence amino-terminal domain and a carboxyl-terminal module with 40-amino-acid, called the SOCS box (4). Among these regions, the SH2 domain is the major functional region and harbors different effects in different SOCS proteins. SOCS box is made of three α-helices and combined with an E3 ubiquitin ligase complex. This structure, together with the E1 ubiquitin-activating enzyme and the E2 ubiquitin-conjugating enzyme, mediates ubiquitination and proteasome degradation of the SOCS binding conjugate (5). Besides, SOCS1 and SOCS3 also possess a special kinase inhibition region (KIR) at the n-terminal, which directly inhibits JAK tyrosine kinase activity (6). The SH2 region in SOCS1 is directly combined with the activation loop of JAK to inhibit the JAK tyrosine kinase activity and the tyrosine phosphorylation of STAT1α (7). After recruiting the SH2 domain to the receptor cytoplasmic domain, SOCS3 exerts inhibition of JAK kinase activity and degradation of the proteasome through the interaction of the SH2 domain and KIR (8).

Currently, SOCS3 has turned out to involve in the process of embryonic development, inflammatory response, immunoregulation, and tumor progression. SOCS3 selectively regulated the signal transduction process of multiple cytokines, including leukemia inhibitory factor (LIF), IL-6, IL-11, G-CSF, and leptin (9). Besides, the loss of SOCS3 in macrophages promoted the activation of STAT and the conversion to the M1 phenotype, improving the phagocytic capacity of macrophages and promoting the differentiation of Th1 and Th17 cells to regulate immune effects (10). Meanwhile, SOCS3 played different roles in different tumor types, being pro-oncogenic or tumor suppressive. In breast cancer, SOCS3 inhibited tumor metastasis and tumor cell proliferation regulated by multiple non-coding RNA (11). In lung cancer, SOCS3 inhibited tumor cell proliferation and angiogenesis in small-cell lung cancer cells *via* inhibiting HIF-1α (12) and promoted apoptosis and cell proliferation in non-small-cell lung cancer through its methylation (13). Mouse model research illustrated that SOCS3 mediated the proliferation and hyperplasia of the crypt and the transformation of inflammation into cancer in the colon (14). Altered SOCS3 combined with chemotherapy or targeted therapy has been demonstrated to sensitize tumors and inhibit tumor progression (15). Besides, SOCS3 mimetic have been shown to reduce tumor growth and inhibit lung metastasis in triple negative breast cancer (TNBC) by suppressing the production of inflammatory cytokines (16). Therefore, based on the performance of SOCS3 in regulating cytokine signaling and immune response, it is of clinical application value to investigate the probable mechanism of SOCS3 in immune-related diseases and tumors and to develop mimetic compounds.

However, current research on SOCS3 is mostly focused on immune regulation. The mechanism of SOCS3 regulating tumorigenesis and its involved signaling pathway is relatively complicated. Therefore, it is necessary to summarize the mechanism of SOCS3 in different tumors and conduct further mechanistic exploration. In this study, we explored the expression pattern and prognostic performance of SOCS3 in pan-cancer and its relationship with immune cell infiltration. Besides, we performed immunohistochemistry (IHC) staining in colon cancer patients with lung metastasis to explore the relationship between SOCS3 status and macrophage infiltration and tumor metastasis.

## Materials and methods

### Pan-cancer expression and immune infiltration analysis

The SOCS3 expression and corresponding clinical follow-up information from different tumors were collected from The Cancer Genome Atlas (TCGA, https://portal.gdc.cancer.gov/). Normal tissue data from the GTEx database (http://gtexportal.org) was the normal control. The SOCS3 expression difference between tumor tissues and normal tissues was displayed using a boxplot (R package, ggplot2, v 3.4.0). Clinical follow-up information consisted of overall survival (OS). The relationship between SOCS3 and the prognosis was examined using the Cox proportional hazards regression model (R package, survival, coxph, v 3.2-7) and Kaplan–Meier analysis (R package, survival, survfit, v 3.2-7). Median levels of SOCS3 expression divided patients into low and high expression groups in each tumor and performed differentially expressed genes (DEGs) analysis (R package, TCGAbiolinks, v 2.24.3). Subsequently, we performed gene set enrichment analysis (GSEA) of DEGs for KEGG and Hallmark pathway in the pan-cancer, of which the top ten were shown (R package, clusterProfiler, GSEA, v 4.4.4). Circular barplot displayed the result of enrichment analysis (R package, ggplot2, v 3.4.0). Cancer types and corresponding abbreviations were available in the official documentation (https://www.cancer.gov/types).

TIMER (Tumor Immune Estimation Resource) database (https://cistrome.shinyapps.io/timer/) was used to evaluate the abundance of tumor infiltrating immune cells (TIICs) in different cancer types, including B cells, CD4+ T cells, CD8+ T cells, neutrophils, macrophages, and dendritic cells (17). The ratio of the immune-stromal component in the tumor microenvironment (TME), the tumor mutation burden (TMB), and Microsatellite instability (MSI) were calculated using the Sangerbox online platform (http://www.sangerbox.com/tool) (18). ImmuneScore and StromalScore are the scores of each sample’s immune and matrix components in the TME, respectively, while ESTIMATEScore is the sum of the two, representing the combined ratio of the two components in the TME, which means that the higher score matched the higher the corresponding component in the TME. These scores were also calculated using the Sangerbox online platform. The correlation analysis between EXO5 and immune-gene signatures was performed by the TIGER (Tumor Immunotherapy Gene Expression Resource) database (http://tiger.canceromics.org/#/) (19).

### Patient population and GEO data population

We collected colon cancer patients who had lung metastasis at the First Affiliated Hospital of Zhejiang University from 2010 to 2020. All patients were histopathologically diagnosed by two pathologists and the attending physician. Clinical information was gathered, including gender, age at diagnosis and stage. The Tumor-Node-Metastasis (TNM) -based staging of colon cancers was defined by the new 8th editions of the relevant Union for International Cancer Control (UICC) and American Joint Committee on Cancer (AJCC) publications. Patients without clinical information and pathological section were excluded. The gene expression data and corresponding clinical information of GSE68468, GSE41258, GSE22834, and GSE6988 data sets were collected from GEO data sets (https://www.ncbi.nlm.nih.gov/geo/).

### Immunohistochemistry staining

A total of 32 colon cancer patients who had lung metastasis were included in this study. Normal intestinal mucosal samples from normal adjacent tissue were used as control samples. The CD68, CD163, and SOCS3 status of all primary tumors and metastases and normal intestinal mucosal samples were conducted by IHC staining through standard staining procedures (SOCS3 antibody: ab16030, Abcam, Cambridge, UK; CD68 antibody: ZM0464; ZSGB-BIO, Beijing, China; CD163 antibody: ZM-0428; ZSGB-BIO, Beijing, China). Brownish-yellow cytoplasmic staining was determined to be positive on target tissue cells. Each sample was assessed with a minimum of 500 cells from 5 representative high-power fields (200 ×) and the proportion of positively colored cells was computed. Positive cell density was divided using a four-point method: 1 point for the proportion of positively stained cells < 25%, 2 points for those between 25%-50%, 3 points for those between 50%-75%, and 4 points for those > 75%. The color intensity of positively stained cells was divided according to the three-point method: 1 point for weakly positive cells, 2 points for moderately positive cells, and 3 points for strongly positive cells. The IHC staining score was calculated by multiplying the density value and color intensity of positive cells, and was determined by the four-point method: 0-1, negative; 2-4, weak (+); 5-8, moderate (++); and 9-12, strong (+++). Two pathologists who were blind to the patient’s clinical characteristics underwent independent IHC staining assessment. All patients were divided into two groups based on IHC staining score: low expression (negative and +) and high expression (++ and +++).

### Enrichment analysis using online web tools

DEGs between the low SOCS3 expression group and the high SOCS3 expression group in colon primary tumor and lung metastasis were compared with |log_2_FC| > 1 and P < 0.05, and the shared DEGs were displayed through the heat map (R package, pheatmap, v 1.0.12). STRING database (https://string-db.org/) (20) was used to conduct the relationship between DEGs, and the PPI network was displayed using the Cytoscape 3.7.1 software. DAVID (https://david.ncifcrf.gov/tools.jsp) was used to perform the Gene Ontology (GO) and the Kyoto Encyclopedia of Genes and Genomes (KEGG) enrichment analysis for DEGs (21). The Venn diagram and enrichment factor bubble chart were performed using the OmicStudio tools at https://www.omicstudio.cn/tool.

### Statistical analysis

Pearson’s chi-squared test and student’s *t*-test were used to evaluate the association of CD68, CD163, and SOCS3 status with gender, age at diagnosis, and stage. The student’s *t*-test was used to evaluate the differences in SOCS3 expression of the GEO data set. A P-value <0.05 was considered statistically significant. Data management and analysis were performed using R version 4.2, GraphPad Prism 8.0 Software (GraphPadSoftware, Inc., SanDiego, CA) and SPSS version 19.0 (SPSS Inc., Chicago, IL, USA). All methods were carried out by relevant guidelines.

## Results

### Pan-cancer expression and prognostic performance of SOCS3

**Figure 1:**
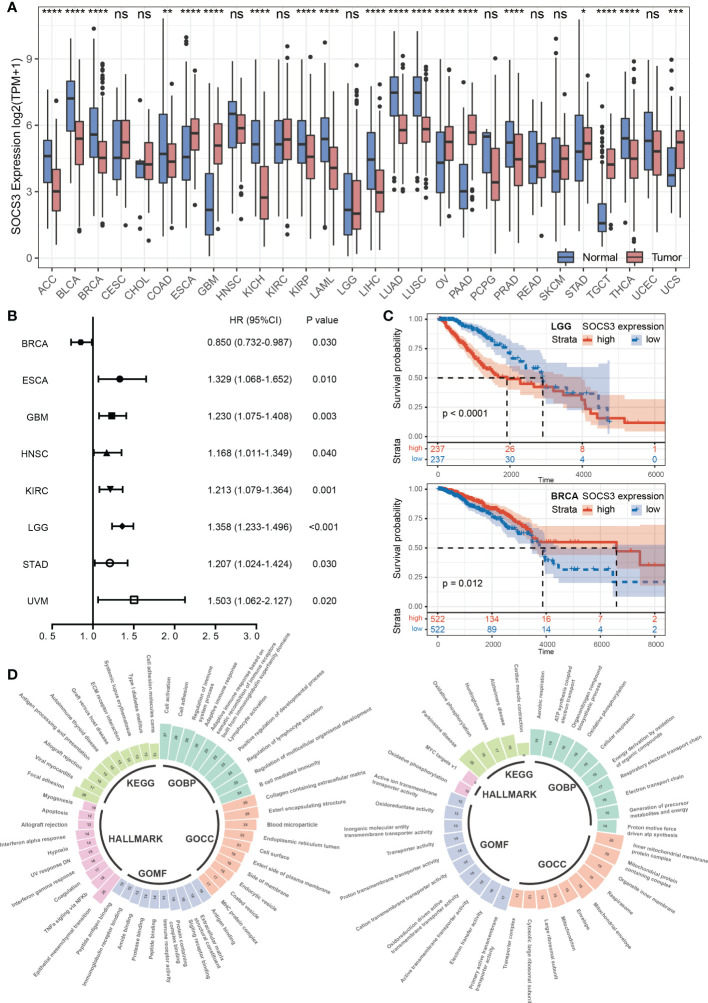
Pan-cancer expression pattern and prognostic performance of SOCS3. **(A)** The SOCS3 expression difference between cancer and corresponding non-tumor normal tissues in different tumors. *P < 0.05, **P < 0.01, ***P < 0.001, ****P < 0.0001. **(B)** The Cox proportional hazards regression analysis of SOCS3 in different tumors. **(C)** The Kaplan–Meier analysis in LGG and BRCA. **(D)** The consolidation of enrichment analysis of GO, KEGG and Hallmark pathway between the low SOCS3 group and high SOCS3 group in different tumors. The expanded form of "ns" is "no significance.

To figure out the difference in SOCS3 expression between normal tissue and tumor tissue in different cancer types, we explored the expression data in TCGA and GTEx databases. The results indicated that urologic neoplasms (including BLCA, KICH, KIRP) and respiratory tumors (including LUAD and LUSC), and ACC, LAML, and THCA were more inclined to low SOCS3 expression, whereas reproductive system neoplasms (including OV, TGCT, UCS) and gastrointestinal cancer (including ESCA, STAD, and PAAD) were more likely to high SOCS3 expression. In addition, the expression of SOCS3 was significantly downregulated in PRAD and BRCA, which belong to reproductive system neoplasms, and COAD and LIHC, members of gastrointestinal cancer (**Figure 1A**).

According to the Cox proportional hazards regression model, the SOCS3 expression was less obviously associated with prognosis and tumor progression. High SOCS3 expression patients harbored a poor prognosis in ESCA, GBM, HNSC, KIRC, LGG, STAD, and UVM, whereas harbored a better prognosis in BRCA (**Figure 1B**). Subsequently, we divided patients into low expression group and high expression group by the median of SOCS3 expression, and performed the Kaplan–Meier analysis. The prognosis of the low SOCS3 expression group in LGG and KIRC was significantly better than that of the high SOCS3 expression group, whereas the opposite was in BRCA (**Figure 1C**). However, over time, the prognosis of the two groups converged.

To shed further light on the potential function of SOCS3 in pan-cancer, we divided patients into low and high expression groups in each tumor according to median levels of SOCS3 expression, and performed DEGs analysis. Subsequently, we performed GSEA through the GO functional annotation, KEGG pathway and the Hallmark pathway and summarized the pathways that were common to different tumors in the enrichment analysis results (**Figure 1D**). For GO enrichment analysis, the high SOCS3 expression group was chiefly enriched in developmental regulation, regulation of cell differentiation, and immune response, whereas the low expression group was in organonitrogen compound biosynthetic process and oxidative phosphorylation. The results of the KEGG enrichment analysis revealed that patients with high SOCS3 expression mainly exhibited enrichment of the ECM receptor interaction, immune-related disease, and antigen processing and presentation. Those patients showed enrichment of epithelial mesenchymal transition, TNFα signaling *via* NFκb, and interferon response for the Hallmark pathway. Therefore, we speculated that there was a possible association between SOCS3 and the immune response and microenvironment in different tumors.

### Relationship between SOCS3 expression and immune response

**Figure 2:**
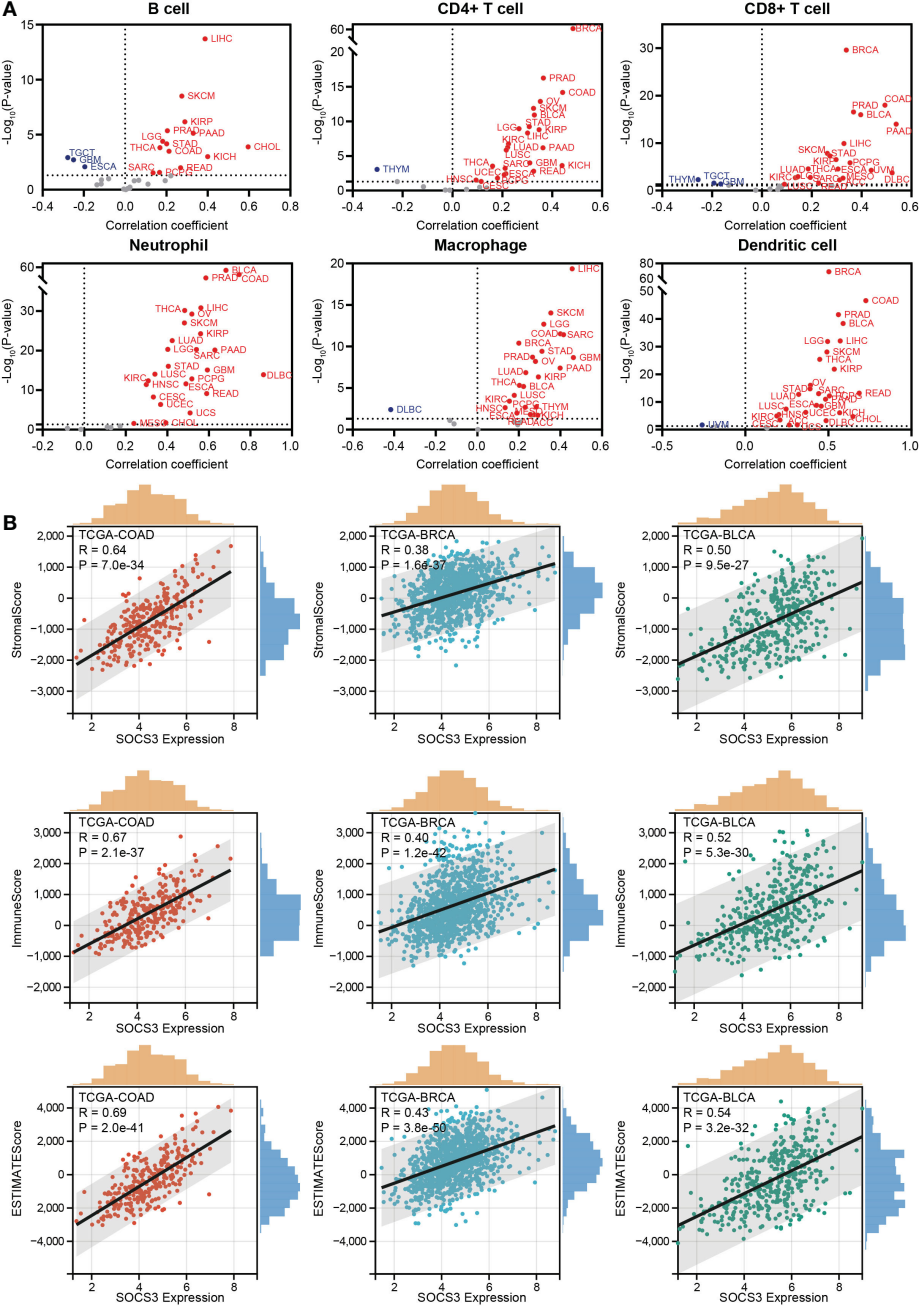
Relationship between SOCS3 expression and immune response. **(A)** The correlation between SOCS3 expression and main immune cell infiltration in different tumors, including B cell, CD4+ T cell, CD8+ T cell, neutrophil, macrophage, and dendritic. Red point: a positive correlation. Blue point: a negative correlation. **(B)** The 3 most significant related tumors in StromalScore, ImmuneScore, and ESTIMATE score.

Based on the above discovery, we explored the relationship between SOCS3 expression and immune cell infiltration in different tumors through the TIMER database, including B cell, CD4+ T cell, CD8+ T cell, neutrophil, macrophage, and dendritic. It is observed that a large scale of tumors was positively related to immune cell infiltration, in which the four most significantly associated tumors were BRCA, LIHC, PRAD, and COAD (**Figure 2A**). Through the ESTIMATE algorithm, immune scores were calculated in different tumors (**Figure 2B**), for which the top three significantly associated tumors were BLCA (R= 0.54, P< 0.001), BRCA (R= 0.43, P< 0.001), and COAD (R= 0.69, P< 0.001).

**Figure 3:**
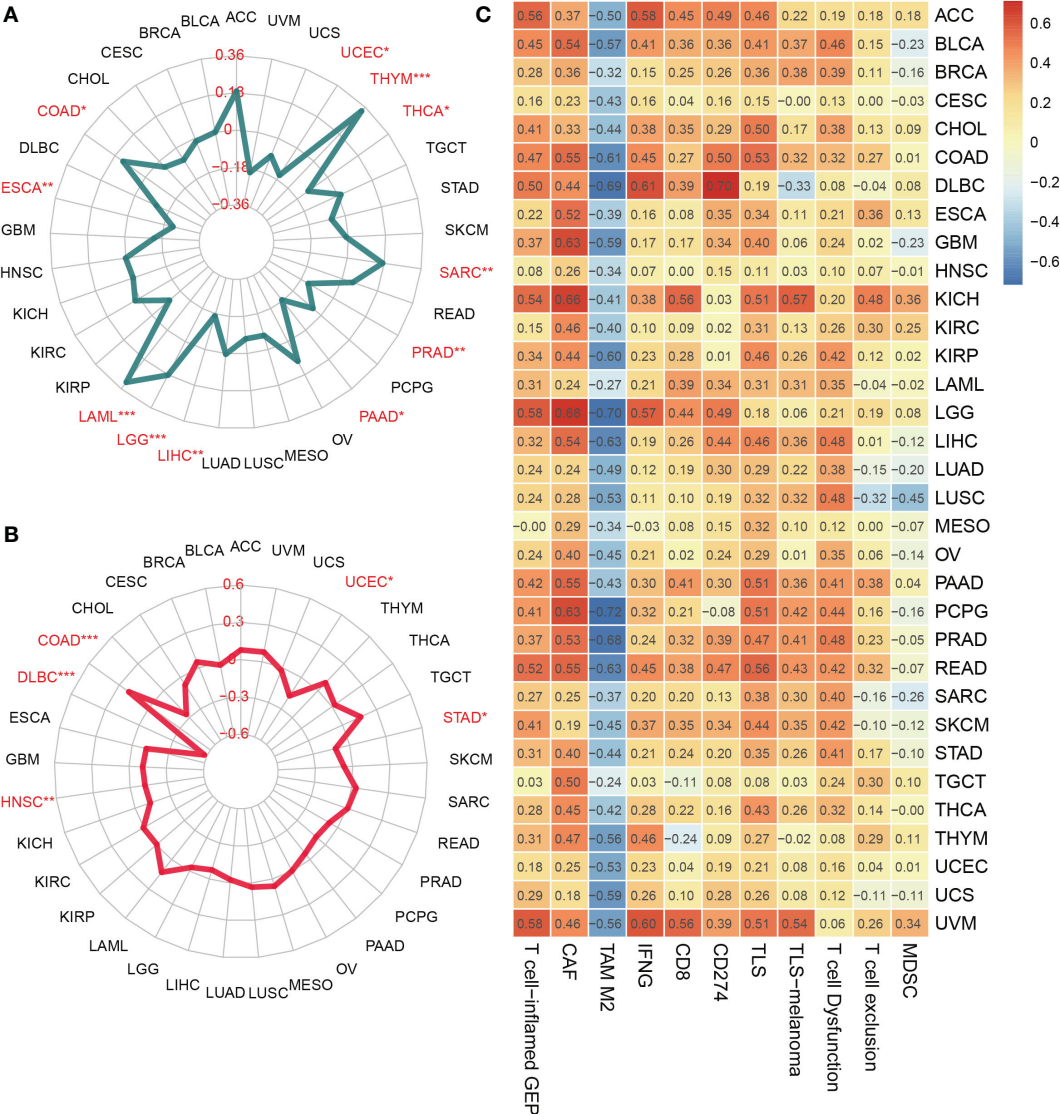
Relationship between SOCS3 expression and tumor mutational burden (TMB), microsatellite instability (MSI) and immune-gene signatures. **(A)** TMB. **(B)** MSI. **(C)** immune-gene signatures. The meaning of the symbol: *P < 0.05, **P < 0.01, ***P < 0.001.

Besides, TMB and MSI, two biomarkers associated with the immunotherapy response, were analyzed concerning the SOCS3 expression in different tumors. The expression level of SOCS3 positively and significantly correlated with TMB in several tumors, including COAD, LAML, LGG, SARC, and THYM, whereas negatively and significantly correlated with that in UCEC, ESCA, LIHC, PAAD, PRAD, and THCA (**Figure 3A**). COAD exhibited positive correlations between SOCS3 expression and MSI, and DLBC, HNSC, STAD, and UCEC exhibited negative correlations (**Figure 3B**). Subsequently, we analyzed the relationship between SOCS3 expression and 11 published immune-gene signatures, including T cell-inflamed GEP, CAF, TAM M2, IFNG, CD8, CD274, TLS, TLS-melanoma, T cell Dysfunction, T cell exclusion, and MDSC. We found that the SOCS3 expression in the majority of tumors positively correlated with immune-gene signatures, except for TAM M2 (**Figure 3C**).

### SOCS3 status in colon primary tumor and lung metastasis and its association with macrophage marker

**Figure 4:**
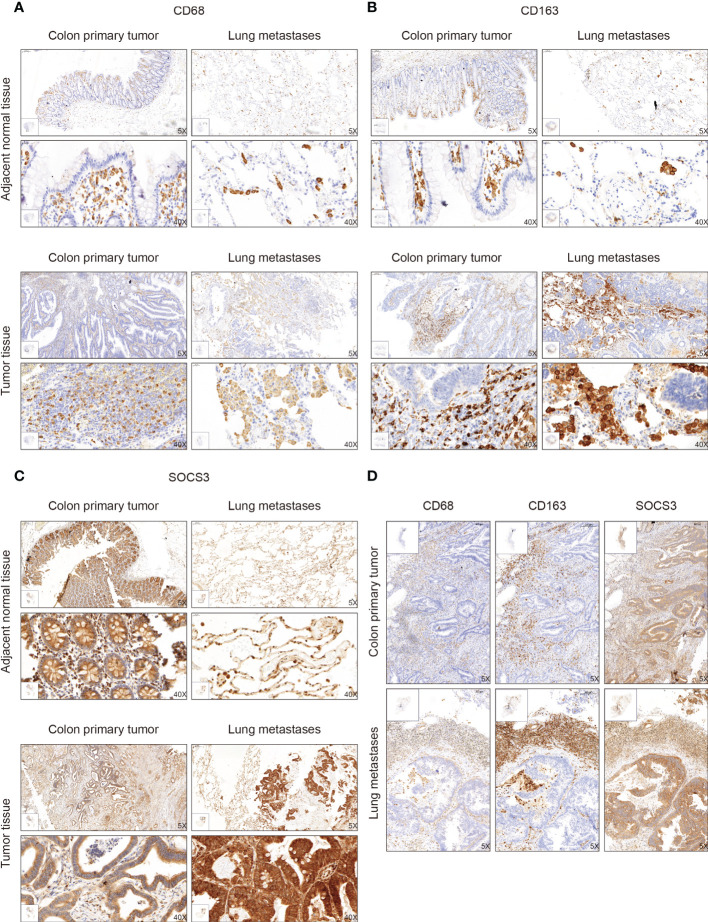
The immunohistochemistry (IHC) staining of CD68, CD163, and SOCS3 between tumor tissue and adjacent normal tissue in colon primary tumor and lung metastasis. **(A)** CD68. **(B)** CD163. **(C)** SOCS3. **(D)** Comparison at the same location.

The function of SOCS3 is mainly focused on immune response, which can regulate macrophage and TH cell functions. Also macrophages in tumors can promote cancer development and metastasis through various mechanisms. Therefore, based on the previous research and the distinct relationship between SOCS3 status and the immune response in COAD, we collected tumor tissue and adjacent normal tissue from 32 colon cancer patients who had lung metastasis, and performed IHC staining with several markers, including CD68, CD163, and SOCS3. CD68 and CD163 are currently the most widely used macrophage marker and are predominantly located in cell membranes and lysosomal membranes. IHC staining indicated that there was a clear aggregation distribution effect on the leading edge of the colon primary tumors and lung metastasis focus for CD68 and CD163 markers, and this effect was more remarkable in lung metastasis than in colon primary tumor (**Figures 4A, B**). Compared with adjacent normal intestinal mucosal tissue, SOCS3 status in colon primary tumor tissue was slightly weak, mainly with moderate positive staining in the cytoplasm (**Figure 4C**). Nevertheless, SOCS3 and CD163 staining in lung metastasis showed more intense membrane and cytoplasm staining than in colon primary tumors (**Figure 4D**).

To search out the relationship between SOCS3 expression and clinical characteristics, we gathered clinical information from these patients during the first visit and calculated the IHC staining score of SOCS3. Patients who had lymph node metastasis and distant metastasis at the initial diagnosis preferred high SOCS3 expression in lung metastasis focus, whereas had no significant difference in colon primary tumors focus (**Table 1**). Besides, the high IHC staining score of SOCS3 and CD163 mostly correlated with lung metastasis focus (**Table 2**). Compared with the colon primary tumor, lung metastasis harbored higher CD163 and SOCS3 expression (**Table 3**). Subsequently, we analyzed the correlation between SOCS3 expression and macrophage infiltration, and found that SOCS3 expression significantly correlated with macrophage markers and high SOCS3 expression was more inclined to high CD163 expression in lung metastasis (**Table 4**). The results suggested that SOCS3 might be associated with macrophage infiltration and tumor invasion in lung metastasis.

**Table 1:** Correlation between clinical characteristics and status of CD68, CD163, and SOCS3 in colon primary tumor.

Colon primary tumor	Cases	CD68 low	CD68 high	P-value	CD163 low	CD163 high	P-value	SOCS3 low	SOCS3 high	P-value
Gender	32			0.673			0.888			0.034
Male	21	18	3		12	9		7	14	
Female	11	10	1		6	5		8	3	
Age(year)										
Median(arrange)	56.5(24-76)									
< 56	16	14	2	1.0	9	7	1.0	9	7	0.288
≥56	16	14	2		9	7		6	10	
TNM stage										
T							0.457			0.080
T1-2	9	9	0		6	3		2	7	
T3-4	23	19	4		12	11		13	10	
N				0.882			0.457			0.337
N0	9	8	1		6	3		3	6	
N1-2	23	20	3		12	11		12	11	
M				0.341			0.556			0.513
M0	13	10	3		6	7		7	6	
M1	19	18	1		12	7		8	11	

**Table 2:** Correlation between clinical characteristics and status of CD68, CD163, and SOCS3 in lung metastasis.

Lung metastasis	Cases	CD68 low	CD68 high	P-value	CD163 low	CD163 high	P-value	SOCS3 low	SOCS3 high	P-value
Gender	32			0.968			0.652			0.593
Male	21	19	2		6	15		4	17	
Female	11	10	1		4	7		3	8	
Age(year)										
Median(arrange)	56.5(24-76)									
< 56	16	14	2	0.544	5	11	1.0	2	14	0.2
≥56	16	15	1		5	11		5	11	
TNM stage										
T				0.833			0.491			0.327
T1-2	9	8	1		2	7		3	6	
T3-4	23	21	2		8	15		4	19	
N				0.833			0.874			0.053
N0	9	8	1		3	6		4	5	
N1-2	23	21	2		7	16		3	20	
M				0.787			0.026			0.060
M0	13	12	1		3	10		5	8	
M1	19	17	2		12	7		2	17	

**Table 3:** Comparison of the IHC staining score for CD68, CD163, and SOCS3 between colon primary tumor and lung metastasis.

	Colon primary tumor (Cases, %)	Lung metastasis(Cases, %)	P-value
CD68			0.689
high	4 (12.5)	3 (9.4)	
low	28 (87.5)	29 (90.6)	
CD163			0.044
high	14 (43.8)	22 (68.8)	
low	18 (56.2)	10 (31.2)	
SOCS3			0.035
high	17 (53.1)	25 (78.1)	
low	15 (46.9)	7 (21.9)	

**Table 4:** Correlation between SOCS3 expression and macrophage markers in colon primary tumor and lung metastasis.

	SOCS3					
	CD68	Low(%)	High(%)	Total(%)	P	R
Colon primary tumor	Low	13 (46.4)	15 (53.6)	28 (87.5)	0.893	-0.024
	High	2 (50.0)	2 (50.0)	4 (12.5)		R-P
	Total	15 (46.9)	17 (53.1)	32		0.898
	CD163	Low(%)	High(%)	Total	P	R
	Low	7 (38.9)	11 (61.1)	18 (56.3)	0.305	-0.181
	High	8 (57.1)	6 (42.9)	14 (43.8)		R-P
	Total	15 (46.9)	17 (53.1)	32		0.32
	CD68	Low(%)	High(%)	Total	P	R
Lung metastasis	Low	6 (20.7)	23 (79.3)	29 (90.6)	0.614	-0.089
	High	1 (33.3)	2 (66.7)	3 (9.4)		R-P
	Total	7 (21.9)	25 (78.1)	32		0.628
	CD163	Low(%)	High(%)	Total	P	R
	Low	5 (50.0)	5 (50.0)	10 (31.3)	0.009	0.459
	High	2 (9.1)	20 (90.9)	22 (68.8)		R-P
	Total	7 (21.9)	25 (78.1)	32		0.008

### SOCS3 expression of the colon primary tumor and metastasis in the GEO database and its enrichment analysis

**Figure 5:**
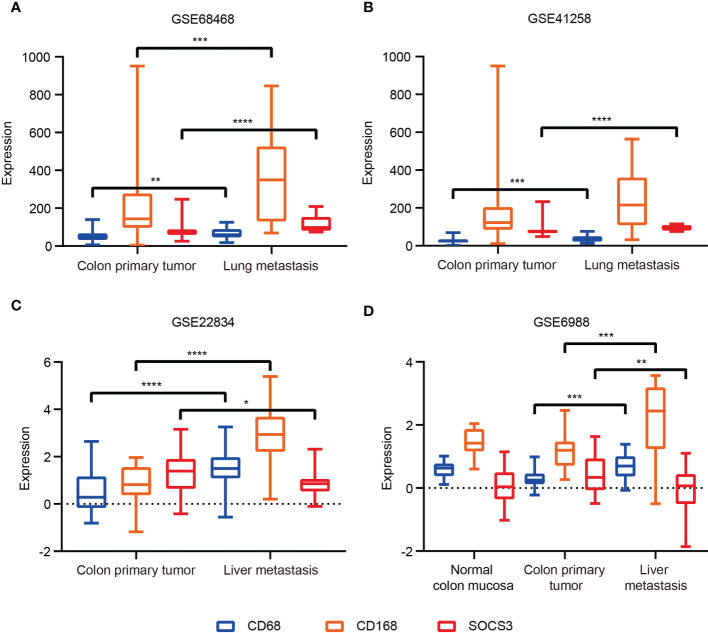
The SOCS3 expression difference between colon primary tumor and metastasis in the GEO database. **(A)** GSE68468; **(B)** GSE41258; **(C)** GSE22834; **(D)** GSE6988. *P < 0.05, **P < 0.01, ***P < 0.001, ****P < 0.0001.

Based on IHC staining results, we investigated transcription data in the GEO database to explore whether the transcript level and protein level have the same characteristics. The most common site of blood metastasis of colon cancer is the liver, followed by the lung, brain and bone. Transcript data sets consisting of the colon primary tumor and metastasis in the GEO database were used, including GSE68468 and GSE41258 (colon primary tumor and lung metastasis), GSE22834 and GSE6988 (colon primary tumor and liver metastasis). The CD68 and CD163 expression levels in metastasis were higher than in the colon primary tumor. The SOCS3 expression of lung metastasis was significantly higher than in the colon primary tumor, whereas that in liver metastasis was the opposite (**Figure 5**). These results revealed that SOCS3 shows different manifestations in different metastasis.

**Figure 6:**
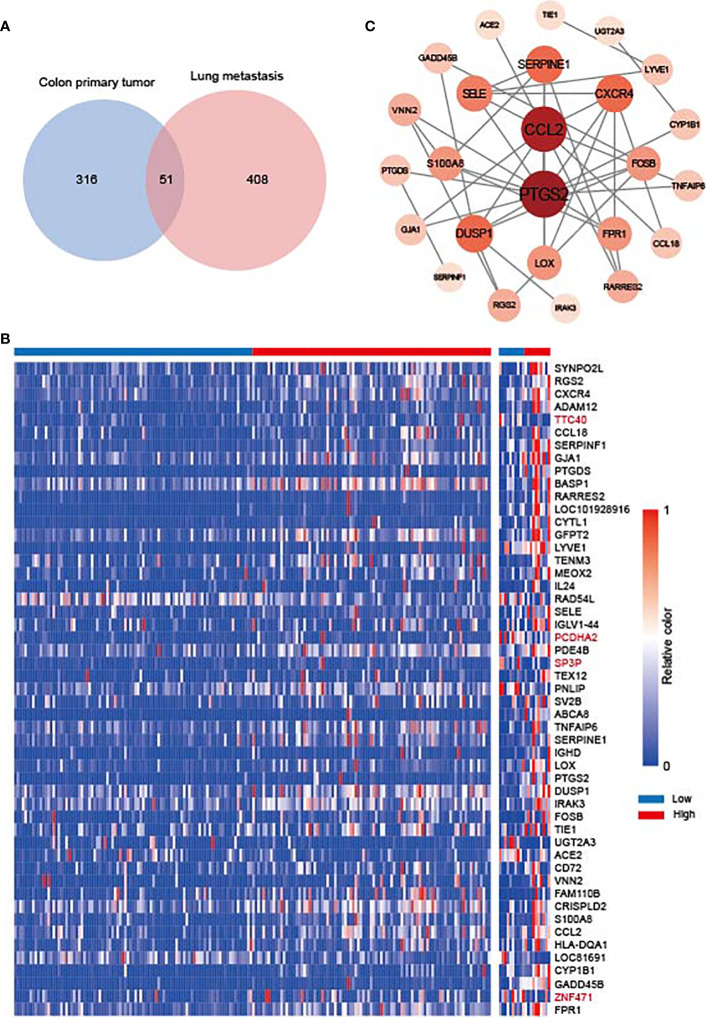
The gene difference in colon primary tumor and lung metastasis. **(A)** Venn diagram for differentially expressed genes (DEGs) between colon primary tumor and lung metastasis in the GSE68468 dataset. **(B)** Heat map of 51 shared DEGs. **(C)** Interplay network of DEGs and two hub genes through STRING and Cytoscape.

**Figure 7:**
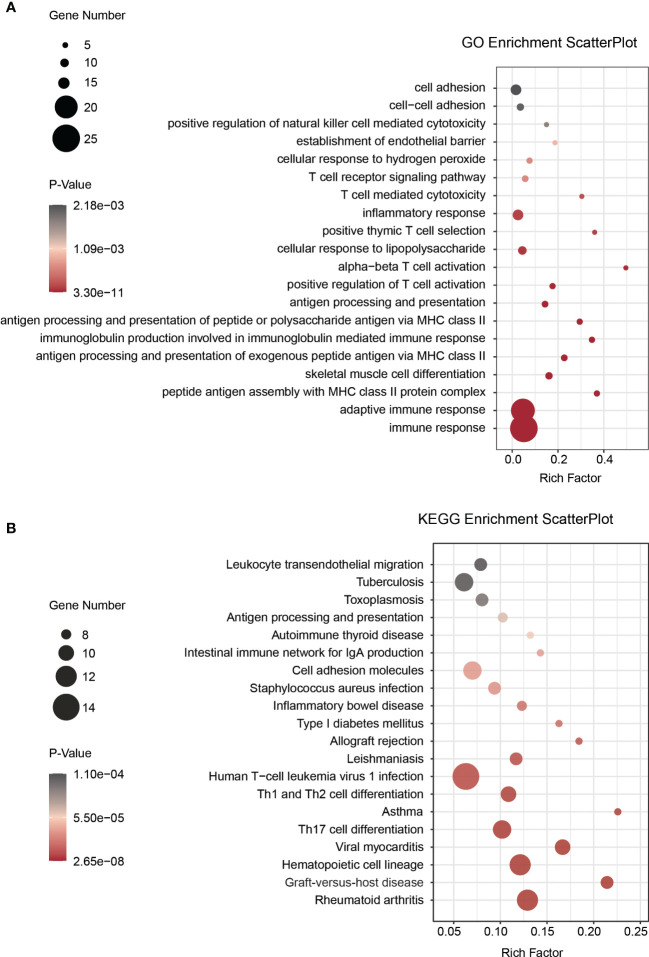
The enrichment analysis between the low SOCS3 expression group and the high SOCS3 group in the exceptional DEGs in lung metastasis. **(A)** Biological process. **(B)** KEGG pathway.

To explore the molecular mechanisms of SOCS3 in lung metastasis, we divided the colon cancer and lung metastasis patients into two groups in the GSE68468 dataset respectively *via* the median of SOCS3. Subsequently, DEGs were compared between low SOCS3 expression and high SOCS3 expression in colon primary tumor and lung metastasis with |log_2_FC| > 1 and p < 0.05. 367 DEGs were screened out in colon primary tumors and 459 DEGs in lung metastasis, and 51 genes were shared by two sets of DEGs (**Figure 6A**). There were 353 upregulated genes and 4 downregulated genes in the colon primary tumor, and 247 upregulated genes and 212 downregulated genes in lung metastasis (**Figure 6B**). Interestingly, four genes that were originally highly expressed in the high SOCS3 expression group in colon cancer became low expressed after lung metastasis, including TTC40, PCDHA2, SP3P and ZNF471. Through STRING and Cytoscape, we conducted the interplay network of 51 shared DEGs and screened out two genes as hub genes, including CCL2 and PTGS2 (**Figure 6C**). Furthermore, the enrichment analysis of GO and KEGG pathways was performed to explore the underlying interplay of the exceptional DEGs in lung metastasis. For GO analysis, 20 biological processes (BPs) were enriched, such as immune response, MHC class II protein related process, antigen processing, T cell activation, and inflammatory response (**Figure 7A**). Regarding the KEGG pathway analysis, Rheumatoid arthritis, Th17 cell differentiation, and Graft−versus−host disease were enriched (**Figure 7B**). These results showed that immune responses and regulations were significantly enriched in both GO and KEGG analysis.

## Discussion

Previous studies showed the abnormal expression of SOCS3 is widely involved in the pathogenesis of various tumors, including migration, cell growth, immune infiltration, inflammation response, and cell death related to various kinds of cytokines (22). Our study comprehensively investigated the expression and potential functions of SOCS3 in different tumors. We found that SOCS3 participated in the occurrence and development of a majority of tumors and presented different patterns in diverse tumors. SOCS3 referred to the prognosis and disease progression of a small part of tumors, and its high expression was more inclined to poor prognosis. Meanwhile, we also found that SOCS3 was positively correlated with main immune cell infiltration in almost each cancer type, especially in COAD. SOCS3 was a predictive marker for poor prognosis in central nervous system tumors and several gastrointestinal cancers, including ESCA and STAD. In COAD, LIHC and PRAD, the SOCS3 expression was significantly positively related to multiple immune cell infiltration. These results indicated that SOCS3 might be a potential target for immunotherapeutic intervention. However, patients with high SOCS3 expression in BRCA have a better prognosis and high immune cell infiltration. This result was also consistent with previous studies that SOCS3 inhibits breast cancer progression and metastasis (11, 16).

Macrophages played an important role in response to tumor immune through two different polarized statuses, including M1 macrophage, promoting tumor cell death *via* interleukin (IL) 12, and M2 macrophage, promoting tumor progression *via* IL10 (23). Suppression of SOCS3 in macrophages exerted anti-inflammatory and anti-tumor effects through the hyperactivation of STAT3 in the myeloid cell (24). Therefore, SOCS3 was recognized as an important feedback regulator mediating the macrophages’ activities and functions (25, 26), and its peptidomimetic was a potential treatment to activate inflammatory cytokines in diseases (27). Based on existing studies of SOCS3 in immune response and its prominent association with immune response in COAD, we explored SOCS3 status and macrophage infiltration in colon cancer patients and the differences in the primary focus and lung metastases of colon cancer. Compared with the colon primary tumor, lung metastasis harbored higher CD163 and SOCS3 expression, and high SOCS3 expression was more likely to be associated with high CD163 expression in lung metastasis. These suggested that SOCS3 might be involved in the process of lung metastasis in colon cancer patients through its interaction with macrophages. Differential analysis and enrichment analysis of colon primary tumor and lung metastasis also suggested that immune response and Th cell activation were involved in this process. Interestingly, SOCS3 showed two completely different trends in liver metastases and lung metastases in colon cancer patients. Also COAD and READ were significantly different both in differences between tumor and adjacent normal tissue and prognosis, and in relationship with immune cells and mutation. This might suggest that SOCS3 had different functions in intestinal tumors with different anatomical locations and might influence the anatomical location of metastasis. Further diverse samples are required to validate these findings and relevant experiments were carried out to investigate the probable mechanism.

However, there are several shortages in our study. Firstly, there was a data deviation due to a retrospective study based on the public database. Patients in the TCGA database were dominated by white people and massive missing data in clinical information might affect the analytical result. Besides, colon cancer samples in our study were relatively small and only performed IHC staining analysis, as well as lacking corresponding follow-up information. Furthermore, we need further a corresponding experiment to certify our findings. For the next step, we will collect more colon cancer and rectal cancer samples and corresponding metastatic tissues at different sites to perform transcriptomic and proteomic analysis and investigate the pattern of other immune cell infiltration. Meanwhile, we will explore the effect of SOCS3 alterations in tumor cells on surrounding macrophage polarization and its role in tumor invasion.

Taken together, our study comprehensively investigated the expression pattern, prognosis performance and relationship with immune cell infiltration of SOCS3 in pan-cancer, and showed that SOCS3 possessed value as a prognostic marker and target for immunotherapeutic intervention in different tumors. Meanwhile, our research gave a new perspective on the relationship between SOCS3 status and macrophage infiltration in colon cancer patients with lung metastasis, which provided support for the possibility of SOCS3 as a potential target of tumor progression and tumor immunotherapy in colon cancer.

## Data availability statement

The original contributions presented in the study are included in the article/supplementary materials, further inquiries can be directed to the corresponding author/s.

## Ethics statement

The studies involving human participants were reviewed and approved by The Ethics Committee of the First Affiliated Hospital of Zhejiang University School of Medicine (the ethics approval number: IIT20210083B). Written informed consent for participation was not required for this study in accordance with the national legislation and the institutional requirements.

## Author contribution

XS and GT designed the research study. XL, ZY, and BC performed the data analysis and interpretation. XL, ZY, LG and BC performed the IHC staining. XL, ZY, LG and BC performed the Statistical analysis. XL wrote the original draft. XL, ZY, LG and BC performed the writing review and editing. All authors contributed to the article and approved the submitted version.
